# Associations between Social Experiences and Psychological Health for Autistic Youth with Low IQ

**DOI:** 10.1007/s10803-024-06378-3

**Published:** 2024-05-04

**Authors:** Julie Lounds Taylor, Virginia Sullivan, Somer L. Bishop, Shuting Zheng, Ryan E. Adams

**Affiliations:** 1 Vanderbilt University Medical Center, PMB 40 – 230 Appleton Pl, Nashville, TN 37203 USA; 2 University of California, San Francisco, USA; 3 Cincinnati Children’s Hospital Medical Center, Cincinnati, USA

**Keywords:** Autism, Social activities, Intellectual disability, Youth, Quality of life, Depression, Peer victimization

## Abstract

**Purpose:**

Social experiences are consistently associated with psychological health among autistic individuals. However, most extant studies on this topic exclude individuals with autism who have lower IQ or are otherwise unable to self-report. The current study addresses this gap by examining associations of negative peer experiences and social participation with psychological health among autistic youth with low IQ.

**Methods:**

An online survey was collected from 268 parents of autistic adolescents and adults ages 15–25. Negative peer experiences included measures of peer victimization and being ignored. Social participation was assessed by the amount of participation and parents’ perceptions of whether their youth felt the amount of participation was meeting their needs. Psychological health was assessed by parents’ report of their youth’s psychological quality of life, as well as whether they felt their son/daughter was currently depressed.

**Results:**

Results suggested low rates of social participation in this sample, with relatively high rates of being ignored. Regression analysis found that lower rates of peer victimization and more activities in which parents perceived that the amount of time was meeting their youth’s needs was associated with higher psychological quality of life and lower likelihood that parents felt their son/daughter was depressed.

**Conclusion:**

Though youth with autism and low IQ are often excluded from interventions aimed at improving social experiences, these findings suggest that promoting positive social experiences and ameliorating negative ones might be an avenue to improving psychological health in this group.

Despite the growing body of autism research, there remains a significant gap in our understanding of the experiences of individuals with autism^1^ and intellectual disability and/or limited language (Jack & Pelphrey, 2017; Russell et al., 2019; Tager-Flusberg & Kasari, 2013; Tager-Flusberg et al., 2016). The 2021–2023 Interagency Autism Coordinating Committee’s Strategic Plan for Autism Research, Services, and Policy emphasized the need to include autistic adults with intellectual disability in research to gain a comprehensive understanding of the spectrum of autism and develop effective interventions for this group (Interagency Autism Coordinating Committee, September 2023). Yet individuals with intellectual disability generally remain excluded from autism research, and particularly from research on psychological health, which limits our understanding of their needs and experiences. For example, a recent review of quality of life studies in autism found that less than 15% of the reviewed studies (4 out of 28) included individuals with autism and intellectual disability (Skaletski et al., 2021).

Systematic exclusion of autistic individuals who are minimally verbal or who have low IQ from research is particularly problematic given the need for studies that inform interventions and supports for this group. Even when researchers who exclude autistic people with intellectual disability are clear that their findings may not apply to this group, subsequent citations of that work nearly always generalize findings to the entire autism spectrum (Russell et al., 2019). Overgeneralizing research findings to individuals with intellectual disability when they were not included in the research studies increases the likelihood that resources will be put toward interventions and supports that have not been shown to be effective or helpful for these individuals. Examining factors that are associated with valued outcomes, like psychological health, in people with autism and intellectual disability is a critical step in better understanding how to improve outcomes in this underserved group.

In the present study, we use the term “psychological health” to indicate absence of mental health challenges (for this study, absence of depression) and positive subjective quality of life. Poor psychological health is a persistent issue in the autism population, with elevated rates of depression (Hudson et al., 2019; Smith & White, 2020) and low subjective quality of life (Ayres et al., 2018; Mason et al., 2018; van Heijst & Geurts, 2015) relative to typically developing peers. Though studies suggest that adults with autism and intellectual disability may be at lower risk for depression than adults with autism without intellectual disability (Hudson et al., 2019), rates remain high in this group, with one population-based study finding that over 35% of adolescents and adults with autism and intellectual disability had scores indicating depression (Bakken et al., 2010) – a rate over two times greater than a comparable sample of individuals with intellectual disability (but not autism). Similarly, low subjective quality of life has been observed for autistic adults who do *and* do not have intellectual disability (Lord et al., 2020). In sum, these studies suggest that even though they are often excluded from research examining mental health and quality of life, psychological health is nonetheless a concern for autistic adults with intellectual disability.

One avenue to improving psychological health among individuals with autism is to investigate the malleable factors that are associated with it. One such promising set of malleable factors is social experiences. Social experiences, including negative peer experiences and social participation, have strong associations with psychological health among autistic youth without intellectual disability, and may also be important to psychological health for those with intellectual disability.

## Negative peer experiences

The prevalence and correlates of negative peer experiences – particularly peer victimization – among autistic children and youth have been widely investigated in recent years. Studies nearly universally find high rates of peer victimization in this population (for reviews/meta-analysis see Park et al., 2020; Schroeder et al., 2014). For example, Cappadocia and colleagues (2012) found that 77% of parents of children and adolescents with autism reported their son/daughter had been bullied at school within the last month. Similarly, Sterzing and colleagues (Sterzing et al., 2012) reported that nearly 50% of high school students with autism had been bullied the previous school year. Interesting, though the Sterzing and colleagues (2012) study was unable to examine rates of victimization for autistic youth who did versus did not have an intellectual disability, they did compare victimization rates in the autism sample to a sample of youth with intellectual disability (and not autism), finding that rates were similar (Sterzing et al., 2012; confirmed in meta-analyses by Park et al., 2020). Thus, peer victimization is a common experience for autistic youth, as well as those with intellectual disability, and is likely also common among those who have both autism and intellectual disability.

Not only are adolescents and youth with autism at-risk for peer victimization, but peer victimization is strongly associated with psychological health. Specifically, studies suggest that higher rates of victimization are associated with greater internalizing symptoms among children and adolescents with autism (Adams et al., 2014; Cappadocia et al., 2012; Park et al., 2020) and lower subjective quality of life among autistic adults (Hong et al., 2016). Though the research seems clear in establishing a link between negative peer experiences (such as peer victimization) and psychological health, none of these studies focused on autistic individuals with intellectual disability. Thus, the present study fills an important gap in the literature by investigating associations between negative peer experiences and psychological health among youth with autism and low IQ.

## Social Participation

Another aspect of social experiences related to psychological health is social participation. Studies consistently find – from childhood through adulthood – that autistic individuals tend to have far less social engagement than their peers who are not autistic (for reviews, see Petrina et al., 2014; Tobin et al., 2014). For example, Orsmond and colleagues (2013) found that 39% of young adults with autism never spent time with friends, 47% reported that friends never called them, and 48% said that they were never invited to social activities with friends; these rates of non-participation were higher than the other disability groups that they examined (including those with intellectual disability without autism). Similarly low rates of participation have been found for autistic high school students (Shattuck et al., 2011a). For example, DaWalt and colleagues (2020) found that these students averaged eight social interactions at school over a two-week period – less than one per school day. As a result of these many studies demonstrating low rates of participation, interventions have been developed and tested to improve social participation (Giummarra et al., 2022; Tanner et al., 2015). Of note, most studies examining social participation in autistic samples do not include individuals with intellectual disability or do not examine social participation by intellectual disability status. Thus, little is known about the social participation of youth with autism who have intellectual disability.

It is generally assumed that low levels of social participation are problematic for autistic individuals; however, the research evidence is more variable (Cameron et al., 2022). Some studies do indeed suggest that more social participation is associated with better psychological health (e.g., Bailey et al., 2020; Bohnert et al., 2019) whereas other studies do not find an association (e.g., Dovgan & Mazurek, 2019; Taylor et al., 2017), and still others find that, under some circumstances, more social participation can lead to worse psychological health (e.g., Mazurek & Kanne, 2010). Alternatively, a more consistent correlate of psychological health is whether the extent of social participation is meeting an individual’s needs. This assertion is supported by a robust body of work suggesting that loneliness is a key correlate of psychological health among individuals with autism (Grace et al., 2022; Kwan et al., 2020). Further, in a recent study of autistic adults, Adams and colleagues (2023) found that among those who infrequently participated in social activities, elevated depression scores were only experienced if they reported that the amount of time in social activities was “too little.” For those who felt as though the amount of time they were spending in social activities was meeting their needs, there was little association between the amount of time in activities and depressive symptoms. Thus, investigation into associations of social participation with psychological health should also include consideration of whether the amount of participation is meeting a person’s needs.

In sum, little is known about the social lives of youth with autism and low IQ, nor about how social experiences might relate to psychological health in this group. The current study fills this important gap in the research literature by addressing the following questions:

1. What are the social experiences (i.e., negative peer experiences, social participation) of youth with autism and low IQ?
2. Are social experiences associated with demographic and behavioral characteristics of the youth, including whether they are in high school (versus having exited high school), sex, and activities of daily living?
3. Among autistic youth with low IQ, which social experiences are independently associated with psychological health (i.e., parent-reported psychological quality of life and parental perception of depression)?

In this study, we collected parental reports of their youth’s social experiences and psychological health. This is a common practice among samples such as this, in which lower IQ scores and/or language limitations make it difficult (or impossible) to consistently obtain valid self-report information. Difficulties obtaining self-report have led to the exclusion of autistic adolescents and adults with intellectual disability from much of the research on psychological health and social activities (Pallathra et al., 2019; Russell et al., 2019); collecting informant report allows us to begin to address this gap in the literature. Parents of autistic youth – particularly of those with intellectual disability – tend to be highly involved in the lives of their sons and daughters, and thus can provide valid reports (Barker et al., 2014).

## Method

### Participants

The study sample included 268 parents of youth with autism and low IQ, recruited from the Simons Simplex Collection registry (SSC; Fischbach & Lord, 2010), the Simons Foundation Autism Research for Knowledge (SPARK) research match registry (Feliciano et al., 2018), and other research and clinical databases maintained at the authors’ institutions. For this study, we operationalized “low IQ” as IQ scores of 70 or below, consistent with the widely accepted IQ cut-point for intellectual disability (American Psychiatric Association, 2022). All youth had been diagnosed with autism spectrum disorder (ASD). Depending on the database from which they were drawn, the ASD diagnosis was either confirmed at study entry by a clinician utilizing information from well-validated instruments such as the Autism Diagnostic Observation Schedule-2 (Lord et al., 2012), or was made by a community professional and confirmed by parental report (Feliciano et al., 2018; Fombonne et al., 2022). Eligibility criteria for the current study were as follows: (1) youth with autism is between the ages of 15 and 25 at the time of recruitment, which corresponds to the age range specified in the United Nations definition of “youth” (found at www.un.org/en/global-issues/youth); (2) youth has an IQ score of 70 or lower; and (3) parent is able and willing to fill out an online survey.

We relied on IQ scores to define our sample (as opposed to a diagnosis of intellectual disability) because that was available across all our recruitment sources, though in various forms. Some registries had IQ scores available from standardized testing, whereas others included parent-reported IQ range (presumably based on previous standardized testing but not directly confirmed by the research team). Though it would have been ideal to also have standardized adaptive behavior scores or evidence of intellectual disability diagnosis as part of our recruitment criteria, this was not consistently available to us. Research suggests that for most people with autism, standardized adaptive behavior scores fall below standardized IQ scores (Duncan & Bishop, 2015), and thus the vast majority of youth in this sample are likely to have intellectual disability. Demographic characteristics of parent respondents and their autistic sons/daughters are presented in Table 1.

**Table 1:** Demographic characteristics of the sample

	M (range) or % (*n*)
**Parent characteristics**	
Age	50.16 (31.4–81.9)
Gender	
Female	93.3% (250)
Male	6.7% (18)
Race	
White	82.8% (222)
Black or African American	9.3% (25)
Asian	4.1% (11)
Other	1.5% (4)
More than one race	1.9% (5)
Did not report	0.4% (1)
Ethnicity	
Hispanic/Latino	10.4% (28)
Not Hispanic/Latino	89.6% (240)
Highest level of completed education	
Less than high school	3.7% (10)
Received high school diploma (or equivalent)	11.2% (30)
Attended/attending college but have not earned a degree	16.8% (45)
Associate’s degree	13.8% (37)
Bachelor’s degree	31.0% (83)
Master’s degree	17.9% (48)
PhD or professional degree	5.6% (15)
Relationship to autistic youth	
Biological mother	87.7% (235)
Step-mother	0.4% (1)
Adoptive mother	5.2% (14)
Biological father	6.3% (17)
Other (great-aunt/guardian)	0.4% (1)
**Youth characteristics**	
Age	19.22 (14.8–26.1)
Sex	
Female	19.4% (52)
Male	80.6% (216)
Gender	
Female	19.0% (51)
Male	80.2% (215)
Non-binary or other	0.7% (2)
Race	
White	78.0% (209)
Black or African American	9.0% (24)
Asian	3.0% (8)
Native Hawaiian or other Pacific Islander	0.7% (2)
Other	0.7% (2)
More than one race	8.2% (22)
Did not report	0.4% (1)
Ethnicity	
Hispanic/Latino	11.6% (31)
Not Hispanic/Latino	88.4% (237)
Living with parent	
Yes	92.9% (249)
No	7.1% (19)
High school status	
In high school	66.8% (179)
Out of high school	33.2% (89)
Perception of depression	
Parent thinks youth is depressed	10.4% (28)
Parent does not think youth is depressed	67.2% (180)
Parent is not sure if youth is depressed	22.4% (60)

*Note*. There were five participants in which youth sex and gender did not match; one was assigned male at birth and currently identified as female, two were assigned female at birth and currently identified as male, and two were assigned female at birth and currently identified as “non-binary or other.”

### Procedures

Research procedures were approved by the Institutional Review Board at [BLINDED FOR REVIEW], as well as by a community advisory committee for the Simons Foundation. For the SSC and SPARK databases, recruitment emails with a brief study description were sent by SPARK/SSC staff to parents of eligible youth; if parents indicated that they were interested in learning more about the study, they then authorized to have their contact information shared with our study team, who followed up by email with information about the study and a link to the electronic consent form and study survey. For local research and clinic registries, emails with a brief study description were sent by project staff to eligible families who had agreed to be contacted for future research opportunities. Families who responded to the emails received another email with a link to the electronic consent form and study survey. All data were collected via online survey between March of 2021 and May of 2022. The online survey took approximately 60–90 min to complete, and parents were paid $50 for their participation.

In all, study advertisements were sent to 1402 families of autistic youth. Of those, 352 (25.1% response rate) either agreed to share their contact information with the study team (participants from SSC/SPARK) or responded to receive more information about the study (local research and clinic registries). This response rate is typical of studies that utilize online registries (Grosvenor et al., 2023; Williams & Gotham, 2022; Zablotsky et al., 2013). Each of these 352 parents were sent the electronic link with the consent form and online survey, of whom 298 signed the consent form, and 281 either fully or partially completed the survey. The current analytic sample included 268 participants who had complete data on the social experiences and psychological health variables included in this analysis. The 13 parents who were excluded from this analysis due to missing data on variables of interest had marginally lower education levels than those who were included, t (279) = -1.82, *p* = .07, and were less likely to be living with the autistic youth, χ^2^(1) = 4.39, *p* < .05. The groups did not differ on parent age, parent sex, parent race (white versus other) parent ethnicity, youth age, youth sex, youth race (white versus other) youth ethnicity, or whether youth had exited high school.

### Measures

#### Psychological Health

*Psychological quality of life* was measured using the psychological domain of the World Health Organization Quality of Life Scale – Brief Report (WHOQoL-Bref; The WHOQOL Group, 1998). This measure was filled out by parents as a proxy report about their autistic youth. Specifically, parents were asked to “Please answer these questions thinking about how [youth’s name] would respond to them, or how he/she would rate these aspects of his/her quality of life, NOT how you would respond. If you are not sure how [youth’s name] would answer, please provide your best guess.” Studies suggest that proxy reports of quality of life have good correspondence with self-reports for adults with autism and/or intellectual disability (Claes et al., 2012; Hong et al., 2016; Schmidt et al., 2010). Across different developmental conditions, family members have higher self-proxy agreement than professionals or care staff (Rand & Caiels, 2015). The Psychological domain of the WHOQoL-Bref consists of 6 statements on psychological facets of quality of life, such as negative and positive feelings and self-esteem. For the current sample, good internal consistency reliability was found for the psychological domain (alpha = 0.77).

*Parental perception of depression* was measured by asking parents whether they believed their son or daughter was currently depressed. Parents could respond “yes,” “no,” or “not sure.” Responses of “yes” or “no” were included in the analysis.

#### Negative Peer Experiences

Youths’ experiences of being *ignored* were reported by parents using the ignored subscale from the Ostracism Experience Scale for Adolescents (OES-A; Gilman et al., 2013). The ignored subscale contains five items, rated on a 5-point scale (1 = *hardly ever*, 5 = *almost always*). Items were reworded to reflect parents’ report about their youth’s experiences (as opposed to self-report). For example, the item “others ignore me” was changed to “others ignore [youth name].” Excellent internal reliability was found for the ignored subscale in the current sample (alpha = 0.92).

Parents reported the level of *peer victimization* experienced by youth on the 18-item Revised Schwartz Peer Victimization Scale (R-SPVS; Adams et al., 2014). All items were re-worded such that parents reported about their youth’s victimization experiences. Parents indicated how often their youth experienced each indicator of victimization, ranging from 1 = never or almost never to 7 = almost every day. Items from this measure comprise 5 dimensions of peer victimization (verbal, relational, physical, social, and electronic). A total score can be calculated to measure overall peer victimization. For this study, we used an average of all items for the total score, which showed very good internal consistency (alpha = 0.96). Parent report of overall peer victimization on the R-SPVS has shown good correspondence with self-reported scores by autistic adolescents (Adams et al., 2014).

#### Social Participation

Information about the youth’s social and recreational activities was collected from parents using an adapted version of the Social Interests and Habits Questionnaire (SIHQ; Gotham et al., 2014). Seven activities were surveyed. Five of them reflected social experiences: time spent with family; time spent with friends; talking on the phone or texting with friends; emailing, chatting online, or playing videogames with friends; and going to social events (e.g., community groups, birthday parties, dances, church socials). The remaining two items reflected recreational experiences: doing a hobby at home (reading a book, playing videogames, etc.), and doing an activity outside the home (bowling, going to movie, going shopping). Based on previous work, in this analysis we focused on the five items that reflected social experiences (Adams et al., 2023).

For each social activity, two measures were recorded: (1) the amount of time the youth spent in the activity; and (2) the parents’ perceptions of whether the youth felt the amount of time spent in the activity was sufficient. For the amount of time for each activity, participants were asked, “How often does [youth’s name]….” followed by the description of the activity, with the following responses: 0 = never, 1 = a few times a year, 2 = at least once a month, 3 = several times a month, 4 = at least once per week, 5 = several times a week, 6 = every day. For evaluation of time in each activity, participants were asked, “If you had to guess [youth’s name]’s opinion, does [youth’s name] think that the amount of time spent in that activity is: “too much time,” “about the right amount of time,” or “too little time.”

From these responses, two variables were created. The *amount of time spent in social activities* was created by summing the scores for time spent across all five social activities (possible range of 0 to 30). *Perception that the amount of time in social activities was meeting the youth’s needs* was calculated by summing the number of responses across activities in which parents indicated that the son/daughter felt the amount of time was “about right” in that activity (possible range of 0 to 5).

#### Demographic and Behavioral Correlates

Parents were asked their autistic youth’s date of birth which was subtracted from the date parents filled out the survey to generate youth’s age, and whether he/she was in high school or had exited high school. Youth sex was gathered by asking parents their son/daughter’s biological sex at birth (male, female). Though studies have shown greater gender diversity among autistic individuals relative to the general population (George & Stokes, 2017; Strang et al., 2014), in this sample of youth with autism and low IQ, there were only 5 cases where parent-reported current gender did not match sex assigned at birth (of 268, or 1.9% of the sample). For two of these cases, parents reported that their youth’s gender was “non-binary or other.” The number of non-binary/other gender youth was too small for us to include them as a separate group in analyses, and we did not wish to combine them with male or female gender nor exclude them in analyses. Thus, we used sex assigned at birth in the primary analysis (supplemental material presents analytic models with gender instead of sex, excluding the two youth with non-binary or other gender).

Activities of daily living was measured using the 17-item Waisman Activities of Daily Living Scale (W-ADL; Maenner et al., 2013). The W-ADL is a parent-report survey that measures activities of daily living in domains such as dressing, grooming, housework/chores, meal-related activities, and activities outside the home. This parent-report survey has been extensively used among adolescents and adults with autism and has demonstrated good psychometric properties including strong associations with the Vineland Screener Adaptive Behavior Composite Score and the Daily Living Skills subdomain score (Maenner et al., 2013). The total W-ADL score was used in analysis, which in this sample included almost the entire possible range (scores of 4 to 32 out of a possible 0 to 34) and had good internal consistency reliability (alpha = 0.91).

### Data Analysis

Listwise deletion was used to generate the sample of 268 for the current analysis (see methods section for comparison of those from the larger sample who were included in versus excluded from this analysis). For Research Question 1, descriptive statistics were used to examine social experiences among youth with autism and low IQ. Overall scale means, standard deviations, and ranges were examined for peer victimization and being ignored. Frequencies were examined to investigate the amount of time that youth were spending in each social activity, as well as the percentage of youth whose parents perceived they were spending about the right amount, too much, or too little time in each activity. Correlations and independent samples t-tests were used in Research Question 2 to examine bivariate associations between demographic and behavioral variables (in high school/out of high school, sex, and activities of daily living), negative peer experiences (being ignored, peer victimization), and social participation (amount, perception that activities were meeting the youth’s needs).

Research Question 3 examined which social experiences were independently associated with psychological quality of life and parental perception that their youth was depressed. Linear regression analysis was used to investigate independent associations between negative peer experiences (being ignored, peer victimization), social participation (amount, perception that activities were meeting the youth’s needs) and psychological quality of life, accounting for demographic and behavioral indicators (in high school/out of high school, sex, activities of daily living). We also used regression analysis to examine independent associations of social experiences with parental perception of whether their son/daughter was depressed; because parent perception of depression is a dichotomous variable (parents who said they did not know whether their son or daughter was depressed were excluded from this analysis), a logistic regression model was used instead of linear regression (model results did not change when parents who did not know if their youth was depressed were included by combining them with parents who thought their youth was depressed; see supplemental material). The independent variables entered into the model were identical to the model examining predictors of psychological quality of life (i.e., in high school/out of high school, sex, activities of daily living, being ignored, peer victimization, amount of social participation, perception that activities were meeting the youth’s needs).

## Results

### Question 1. Frequency of Social Experiences

Peer victimization scores in this sample covered the entire possible range, from 1 (“never or almost never” for all items) to 7 (“almost every day” for all items). Despite this wide range, the average score for peer victimization was low, averaging 1.62 (falling between “never or almost never” and “once a year”) with a standard deviation of 0.97. Over one-third of the sample (34.7%, *n* = 93) scored a “1”, indicating that they responded “never or almost never” to all the peer victimization behaviors that were queried. Over three-fourths (78.7%, *n* = 211) had an average score of 2 or below; a 2 corresponds to an average item rating of “once a year.” Just under 10% (8.2%, *n* = 22) of autistic youth with low IQ were reported to be victimized “about once a day” (score of 7) for at least one of the victimization items.

Scores on being ignored also covered the entire possible range (5 to 25), but these behaviors, on average, were more frequent and variable in this sample. The mean score was 11.70, with a standard deviation of 5.51. Though 21.3% of the sample scored a “5” indicating “hardly ever” experiencing all of the queried behaviors related to being ignored, one-third (33.2%) of youth had scores placing them in the top one-half of possible scores on the scale (i.e., 15 or higher on a scale of 5 to 25). This stands in stark contrast to the peer victimization scale, in which only 4.1% of youth had scores that fell into the top half of possible scores on the scale. (i.e., 4 or higher on a scale of 1 to 7). About 13% of the sample (13.4%, *n* = 36) received the maximum score on one or more ignore items, indicating that they “almost always” experienced at least one of the indicators of being ignored.

Table 2 presents the frequencies of specific social activities for youth with autism and low IQ. For ease of interpretation, in the table (but not in analyses for Research Questions 2 or 3) the amount of time spent in each activity was condensed to “never” (original score of 0), “sometimes” (original score of 1 to 3, reflecting “a few times a year” to “several times per month”) and “frequent” (original score of 4 to 6, reflecting “at least once a week” to “every day”); frequencies for each response option (not grouped) are available in supplemental material (Supplemental Table 1). As can be seen from Table 2, youth spent the most time with family members, while less time was spent with friends and attending social events. Nearly 40% of parents reported that their son or daughter *never* spent time with friends and 20% reported that their son or daughter never attended social events; only 12% attended social events on a frequent basis. Not surprisingly, given the lower verbal abilities of this sample, social activities with friends that required verbal or written communication were least common, with over 60% of youth never talking on the phone or texting with friends, and over 70% of youth never interacting online with friends. Alternatively, nearly 80% spent time with family members once a week or more (likely reflecting that most youth lived at home). When summing the amount of time across all five variables, we observed a tremendous amount of heterogeneity, with scores ranging from 0 (i.e., no time in any social activity) to 27 (out of a possible maximum of 30).

**Table 2:** Frequency of responses to social participation questions among parents of youth with autism and low IQ

	Amount - % (*n*)			Evaluation - % (*n*)		
Variable	Never	Sometimes (less than once a week)	Frequent (once a week or more)	About the right amount of time	Too little time	Too much time
Spend time with family members	0.7% (2)	19.8% (53)	79.5% (213)	76.5% (205)	15.7% (42)	7.8% (21)
Spend time with friends	38.8% (104)	34.0% (91)	27.2% (73)	54.1% (145)	43.7% (117)	2.2% (6)
Talk on the phone or text with friends	61.9% (166)	16.0% (43)	22.0% (59)	67.5% (181)	29.1% (78)	3.4% (9)
Email, chat online, play videogames with friends	70.9% (190)	11.9% (32)	17.2% (46)	63.1% (169)	32.5% (87)	4.5% (12)
Go to social events (community groups, birthday party, etc.)	20.1% (54)	67.9% (182)	11.9% (32)	52.6% (141)	37.3% (100)	10.1% (27)

We next examined parents’ perception that social activities were meeting their son/daughter’s needs (see Table 2). Parents were most likely to think that their son/daughter would perceive the amount of time with family members to be “about right” and least likely to think their youth would feel the amount of time spending time with friends or going to social events in the community was “about right.” In fact, nearly as many parents reported that their son/daughter would think the amount of time they were spending with friends was too little as those who thought it would be “about right.” Overall, nearly one-half of parents believed that their youth felt they were spending about the right amount of time in four (18.7%, *n* = 50) or all five (30.2%, *n* = 81) of the social activities that were queried. When parents perceived that the amount of time was not “about right,” they were much more likely to report that their son/daughter felt the time was too little as opposed to too much (given the relatively few youth who were reported to be spending too much time in activities, supplemental analyses excluded those youth from the regression models; as can be seen in supplemental material, the pattern of findings was unchanged).

### Question 2. Associations Between Social Experiences and Demographic/Behavioral Variables

Correlations between activities of daily living and social experiences are presented in Table 3. In this same table, we included the Cohen’s *d* effect sizes for independent samples t-tests comparing the other variables by high school exit status and youth sex (as high school exit and youth sex are dichotomous, it would be inappropriate to compute correlations). Youth who were out of high school had higher scores on the ignore scale (M = 12.96, SD = 5.60) compared to those who were in high school (M = 11.07, SD = 5.37), t (266) = 2.67, *p* < .01. Females had higher scores than males on peer victimization. The mean victimization score for females was 1.89 (SD = 1.34) compared to 1.55 (SD = 0.84) for males, t (266) = -2.25, *p* < .05. Youth with greater activities of daily living tended to have more social activities and were less frequently ignored by peers, but they were also victimized more often by their peers. None of the demographic variables examined was associated with parents’ perceptions of whether social activities were meeting the youth’s needs.

**Table 3:** Bivariate associations between youth demographics and social experiences variables

	1.	2.	3.	4.	5.	6.	7.
1. High school status (1 = in high school)	- - -		-0.04	0.35**	-0.06	0.12	-0.05
2. Youth sex (1 = female)		- - -	0.09	0.01	0.35*	0.21	0.00
3. Activities of daily living			- - -				
4. Ignored			-0.13*	- - - -			
5. Peer victimization			0.14*	0.41***	- - - -		
6. Amount of social activities			0.36***	-0.15*	0.17**	- - - -	
7. Perception activities are meeting needs			-0.00	-0.17**	-0.21***	0.03	- - - -

*p < .05 **p < .01 ***p < .001

*Note*. Statistics shown in the bottom triangle are Pearson’s r correlations. Statistics shown in the top triangle are Cohen’s *d* effect sizes for t-tests comparing means by sex and high school status. Sex and high school status were not associated with each other, χ^2^ (1) = 0.32, *p* = .57.

In terms of associations between social experiences, the strongest association was between peer victimization and being ignored (*r* = .41): those who experienced higher scores on victimization tended to also experience higher scores on being ignored. There were significant correlations between peer victimization and being ignored with amount and perception of social activities. Youth with higher victimization scores and lower scores on the ignored variable tended to have more time in social activities. Both higher victimization and higher ignored scores were associated with fewer activities in which parents felt the time spent was meeting their youth’s needs. The association between amount of social activities and number of activities that were meeting the youth’s needs was not statistically significant.

### Question 3. Associations Between Social Experiences and Psychological Health

Multiple regression analyses examined independent associations between negative peer experiences and social participation with psychological quality of life accounting for demographic and behavioral variables. Estimates from the regression model are presented in Table 4. In total, the full model accounted for 22.8% of the variance in psychological quality of life. Youth with lower peer victimization scores, a greater amount of social activities, and more social activities in which parents perceived the amount was meeting their needs had higher scores on psychological quality of life.

**Table 4:** Estimates from regression equation examining demographic variables, negative peer experiences, and social participation as independent predictors of psychological quality of life

Independent Variable	Unstandardized beta	Standard error	Standardized beta
Demographic			
High school status (1 = in high school)	0.74	0.44	0.09
Sex (1 = female)	-0.51	0.52	-0.06
Activities of daily living	0.07	0.04	0.11
Negative peer experiences			
Being ignored score	-0.02	0.04	-0.03
Total peer victimization score	-0.99	0.25	-0.26***
Social participation			
Amount of social activities	0.08	0.04	0.12*
Perception activities are meeting needs	0.65	0.13	0.29***

*p < .05 ***p < .001

**Table 5:** Estimates from logistic regression equation examining demographic variables, negative peer experiences, and social participation as independent predictors of parent perception of depression

Independent variable	Beta	Standard error	Adjusted OR	95% CI for OR
Demographic				
High school status (1 = in high school)	-1.29	0.52	0.28*	0.10–0.77
Sex (1 = female)	-1.51	0.80	0.22	0.05–1.06
Activities of daily living	-0.06	0.04	0.94	0.86–1.02
Negative peer experiences				
Being ignored score	-0.10	0.06	0.91	0.81–1.02
Total peer victimization score	1.11	0.27	3.04***	1.78–5.19
Social participation				
Amount of social activities	0.09	0.05	1.09	0.99–1.20
Perception activities are meeting needs	-0.52	0.16	0.59**	0.43–0.81

*p < .05 **p < .01 ***p < .001.

*Note*. OR = Odds Ratio. CI = Confidence Interval.

Logistic regression models were run to test independent associations between the variables listed above and parents’ perception of whether they thought their son or daughter was depressed. Note that for this analysis, we excluded 60 parents who weren’t sure if their son or daughter was depressed, resulting in a sample size of 208. High school exit status, total peer victimization scores, and perception of time spent in social activities were independently associated with whether parents thought their son or daughter was depressed. Parents of youth who were in high school (versus out of high school) and who had more (versus less) social activities that were meeting the youth’s needs were less likely to think that their son/daughter was depressed. Alternatively, parents of youth with higher peer victimization scores were more likely to think that their youth was depressed (Table 5).

## Discussion

Findings from this study suggest that, among autistic youth with low IQ, both negative peer experiences and social participation are associated with psychological health. Importantly, this study included both positive and negative indicators of psychological health, as well as positive and negative indicators of social experiences. Thus, it was not just the absence of negative social experiences that was associated with psychological health (including both lower likelihood of parental perception of depression and higher psychological quality of life), but also the presence of more social interactions that were perceived to be meeting the youth’s needs. Though caution is indicated given the cross-sectional nature of the study, these findings suggest that both reducing negative social experiences *and* increasing positive social experiences may be important for promoting psychological health among youth with autism and low IQ.

Relative to peer victimization, being ignored by peers was a more frequent occurrence among autistic youth with low IQ in this sample. The mean ignore score in this sample was substantially higher than in a sample of adolescents in the general population (11.70 versus 7.1; Bowker et al., 2014), though relatively few youth (13.4%) were reported to be “almost always” ignored. Alternatively, mean peer victimization scores were substantially lower than scores on this same measure in a sample of autistic youth who could self-report (Adams et al., 2014); with the mean score falling between “never/almost never” and “once a year.” Though they appeared to be less frequent in this sample than being ignored, parent-reported peer victimization experiences were related to both indices of psychological health. Thus, it may be useful to closely monitor the psychological health of youth with autism and low IQ when peer victimization does occur.

Consistent with extant research, low rates of social participation were found in this sample of autistic youth with low IQ. Nearly 40% of youth were reported to never have spent time with friends over the past year, and just over 20% were reported to have never gone to a social event during that time. The rate of never spending with friends is almost identical to rates found in the National Longitudinal Transition Study-2, a nationally representative sample of high school (Shattuck et al., 2011b) and post-high school (Orsmond et al., 2013) autistic youth. Importantly, social participation data analyzed from that study were collected in 2001 (for youth in high school) and 2009 (when youth were out of high school) – up to two decades prior to the present study. Thus, despite many efforts aimed at inclusion for youth and adults with disabilities into society, results from the present study suggest that there remains a large group of autistic youth with low IQ who are disconnected from social activities with friends. Time with friends was also the type of social participation that parents in this study most often perceived their youth would like to be spending more time. Thus, not only is the rate of disconnection from friends high, but it is the type of social activity which seems to be the highest unmet need (followed closely by time spent going to social events). Given the associations observed between perception of social activities and psychological health in this study, examining ways to encourage not just social participation but development of friendships among youth with autism and low IQ is likely an important area for future research.

Similar to previous research among autistic adults who could self-report (Adams et al., 2023), the number of social activities that were meeting the youth’s needs was strongly associated with psychological health (and more consistently associated than the amount of participation). This finding suggests a number of potential avenues that could be explored in interventions to improve psychological health among autistic youth with low IQ. It may be that a behavioral activation approach – an effective treatment for depression in the general population (Ekers et al., 2014; Kanter et al., 2010) – could be effective in decreasing depression among youth with autism and low IQ. Behavior activation, with its focus on behavior change and increased engagement with reinforcing activities, may be more accessible than cognitive behavior therapy to these youth (Bal et al., 2023; Jahoda et al., 2017). Alternatively, it may be that increased engagement in meaningful social activities for autistic youth with low IQ is best achieved by intervening at the community or societal level. Opportunities for meaningful social engagement can be limited for these youth – both in frequency and variety (Budavari et al., 2022; Ratcliff et al., 2018). Policies and practices that facilitate community inclusion across a range of social activities – while also providing the supports and accommodations needed to allow true participation – may be helpful in promoting psychological health. Importantly, our findings suggest that any efforts to increase social participation and engagement should keep a focus on whether the activities are perceived by youth as desirable and meeting their needs.

Individuals with autism and low IQ are often excluded from studies aimed at improving social experiences – especially in adolescence and adulthood. Interventions focused on social relationships among autistic individuals, such as The Program for Education and Environment of Relational Skills (PEERS; Laugeson et al., 2012) and Healthy Relationships on the Autism Spectrum (HEARTS; Rothman et al., 2022), only include autistic youth who can self-report and typically have IQ scores in the average or higher range (Rothman et al., 2022; Zheng et al., 2021). Our findings suggest that those with autism and low IQ would also benefit from programs to improve social functioning, and future research should examine how to develop programs that will meet their needs.

There is one potential caveat to encouraging broader social participation among autistic youth with low IQ suggested by this study. We found that youth who had greater social participation were also somewhat more likely to experience higher levels of peer victimization. Relatedly, youth with higher (versus lower) activities of daily living scores tended to have higher scores on the peer victimization measure. This mirrors associations in broader autism samples between spending more time in general education classes (versus special education) and greater likelihood of peer victimization (Park et al., 2020). Encouraging inclusion of people with disabilities into broader society has been an important focus of advocacy and policy for many years (Patterson, 2018), and benefits individuals with disabilities as well as society. Our findings suggest that programs or interventions aimed at increasing social participation and community integration among youth with autism and low IQ should also be vigilant about the potential for negative peer experiences and build in necessary support to foster safe and positive social experiences.

Finally, these findings add evidence to the rapidly growing body of literature suggesting that the years after high school can be a challenging time for autistic youth. Parents of youth who had exited high school were more likely to think their sons/daughter were depressed relative to parents of youth in high school. This pattern is consistent with longitudinal research suggesting that improvements in internalizing problems observed while youth with autism are in high school slow or even stop after high school exit (Taylor & Seltzer, 2010). We also observed higher scores on the ignored scale for autistic youth who had exited high school relative to youth still in school. Thus, our findings point to the continued need for more research to understand how to improve the transition out of high school and into adult life for autistic youth.

As with any study, there are important limitations of this study that are worth noting. Because many youth in this study were unable to self-report, we relied on parent report for all indicators of social participation and psychological health. Though this choice allowed us to include youth who are often excluded from research due to challenges providing self-report, our measure of perception of social experiences was reliant on parents’ assumptions about how their son or daughter might be feeling about their social participation. Our measures of negative peer experiences – many of which might occur in a school setting – were dependent on parents’ knowledge of whether and how frequently their son or daughter might be victimized and/or ignored. Though this is reason for caution, a meta-analysis did not find a difference between self-report and parent-report of peer victimization among students with autism (Maïano et al., 2016).

Our indicators of psychological health were also dependent on parents’ perceptions of their youth’s psychological health. Indicators of depression are particularly difficult to measure among youth with intellectual disability, as it often goes unrecognized and under-diagnosed (Peña-Salazar et al., 2018; Scott & Havercamp, 2018). Thus, instead of relying on diagnoses of depression (which likely under-represent rates of depression in this group), we focused instead on parents’ perception of whether their son or daughter was depressed. Even this was difficult for many parents to answer, with over 20% of parents in this sample reporting that they were not sure whether their youth was depressed. Future research among autistic youth with low IQ or intellectual disability should consider collecting data on social experiences from multiple reporters (including teachers or other school-based personnel) and alternate measures of psychological health, including questionnaires, clinical interviews, or observations developed specifically for adults with intellectual disability (such as the Anxiety, Depression, and Mood Scale; Esbensen et al., 2003). Perhaps most importantly, research should prioritize developing better tools to gather information about internal states like depression and feelings about social experiences directly from individuals who would be unable to complete currently existing self-report instruments (Schiltz et al., 2024).

By relying on existing databases and registries to recruit our study sample, we were beholden to the racial and ethnic compositions of those registries. The sample of families of youth with autism and low IQ in this study does not represent families who do not have access to the internet, and families from racial and ethnic minoritized groups are under-represented. Future research in this area (and all areas of autism research) needs to make concerted efforts to engage these participants in research so that we can ensure that findings from studies such as this are not over-generalized to populations to which they do not apply.

Finally, the inconsistency of IQ data available to us did not allow for further stratification of the sample by IQ, nor did we have consistent information about adaptive behavior to allow us to determine whether the youth would meet formal diagnostic criteria for intellectual disability. In DSM-5, a standardized IQ score of 70 or below (2 standard deviations below the mean) is one of the criteria for an intellectual disability diagnosis, along with impairments in adaptive functioning (American Psychiatric Association, 2022). Given research showing that standardized adaptive behavior scores often fall well below standardized IQ scores for people with autism (Duncan & Bishop, 2015), the vast majority of youth in this sample are likely to meet diagnostic criteria for intellectual disability. Future research should examine the heterogeneity within those with low IQ or intellectual disability, with more refined stratification at different levels of intellectual disability. It is likely that the social experience of young adults with more severe to profound impairments in cognitive functioning differ dramatically from those with mild intellectual disability or borderline IQ, and it will be important to investigate these within-group differences to better understand how to support those with low IQ.

These limitations are offset by a number of strengths. Instead of focusing only on mental health challenges, this study also considered indicators of positive psychological health. This is consistent with more contemporary conceptualizations of mental health; though mental illness and mental health were in the past conceptualized as two ends of a continuum, research suggests that these are related but distinct concepts (Nes et al., 2013; Routledge et al., 2016). Absence of mental illness symptoms does not guarantee positive psychological health, and thus it is important to understand both. Similarly, instead of focusing only on negative peer experiences, we also considered positive indicators of social experiences. This focus on encouraging the positive in addition to ameliorating the negative is in-line with research and practice recommendations from autistic self-advocates (Benevides et al., 2020; Frazier et al., 2018). Perhaps the most significant strength of this study, however, is its focus on autistic youth with low IQ. These youth are often left out of research in adolescence and adulthood (Interagency Autism Coordinating Committee, September 2023; Russell et al., 2019), as well as from treatment trials (Stedman et al., 2019). Our findings suggest that extension of interventions aimed at promoting positive social experiences (and ameliorating negative ones) may be an important venue to improve psychological health in this group, just as it is for autistic youth with average or above IQ.

## Footnotes

1 The literature suggests that there is variability in preferences for person-first versus identity-first language among individuals on the autism spectrum (Bury et al., 2023). To honor that variability, we use person-first (i.e., autistic youth) and identity-first (i.e., youth with autism) language interchangeably.
